# Optimizing rabbit semen cryopreservation using different cryoprotectants in a Tris-based extender

**DOI:** 10.3389/fvets.2026.1768333

**Published:** 2026-02-05

**Authors:** Wael A. Khalil, Sara F. Fouda, Ibrahim T. El-Ratel, Ehab El-Haroun, Ramya Ahmad Sindi, Mahmoud Moussa, Sameh A. Abdelnour

**Affiliations:** 1 Department of Animal Production, Faculty of Agriculture, Mansoura University, Mansoura, Egypt; 2 Department of Poultry Production, Faculty of Agriculture, Mansoura University, Mansoura, Egypt; 3 Department of Animal, Poultry and Fish Production, Faculty of Agriculture, Damietta University, Damietta, Egypt; 4 Department of Integrative Agriculture, College of Agriculture and Veterinary Medicine, United Arab Emirates University, Al Ain, United Arab Emirates; 5 Department of Clinical Laboratory Sciences, Faculty of Applied Medical Sciences, Umm Al-Qura University, Makkah, Saudi Arabia; 6 Emirates Smart Camel Center, Umm Al Quwain, United Arab Emirates; 7 Department of Theriogenology, Faculty of Veterinary Medicine, Suez Canal University, Ismailia, Egypt; 8 Department of Animal Production, Faculty of Agriculture, Zagazig University, Zagazig, Egypt

**Keywords:** cryoprotectant, disaccharides, oxidative stress, rabbit, sperm cryopreservation

## Abstract

This experiment investigates the potential effects of different cryoprotectant molecules, such as glycerol (GL) and dimethyl sulfoxide (DMSO), added to a Tris-based extender focusing on assessing sperm quality, antioxidant status, acrosome integrity, apoptosis, semen microbiota of post-thawed rabbit semen, and *in vivo* fertility trial in rabbit does. The Tris-based extender was supplemented with various cryoprotectants to form the following five experimental treatments: 4% DMSO (DM4), 4% DMSO+50 mM trehalose (DMTR), 4% DMSO+50 mM sucrose (DMSU), 4% glycerol (GL4), or a mixture of 2% DMSO + 2% glycerol (DMGL2). The results indicate that the DM4 group had better results for progressive motility, viability, and membrane integrity (*p* < 0.001). Sperm kinematic parameters were the greatest in all DMSO groups compared to the GL groups (*p* < 0.01). Fortified Tris with DMTR or DMSU significantly improved live sperm with intact acrosomes and significantly reduced live sperm with detached acrosomes (*p* < 0.01). DMTR group had the greatest total antioxidant capacity (TAC) and lowest malondialdehyde (MDA) and reactive oxygen species (ROS) levels compared to other groups (*p* < 0.01). Moreover, nitric oxide decreased in DMTR or DMSU groups compared to other groups (*p* < 0.05). Viable sperm was the greatest in DMSU group, while apoptotic (%) was the lowest in DMSU and DMTR groups (*p* < 0.01). Total bacterial count was higher in the DM4 group, while lowest in the DMSU group (*p* < 0.01). While the DMSU treatment resulted in the highest conception rate, no significant differences were observed in litter size across groups (*p* > 0.05). The cryo-tolerance of rabbit spermatozoa was significantly improved by modifying the freezing extender to include 4% DMSO enriched with either 50 mM Trehalose or 50 mM Sucrose. This optimized formulation enhanced post-thaw parameters, specifically leading to higher sperm kinematics (e.g., motility and velocity), preserved acrosome integrity, and a reduction in apoptosis-like changes. The mechanism for this protective effect is attributed to the synergistic action of DMSO and the disaccharides in mitigating oxidative stress by enhancing the activity of intrinsic antioxidant defenses within the spermatozoa.

## 1 Introduction

Given the challenge of protein deficiency in developing nations, rabbit breeding presents a particularly advantageous livestock industry for supplying essential animal proteins (1). The rabbit is a worthy animal for both the biomedical sciences and animal protein sources (2). Rabbits are characterized by a high reproductive rate relative to other livestock and are valued not only as an excellent source of dietary protein (3) but also as an optimal model for various research fields, particularly reproductive biology (4). In livestock and animal breeding, cryopreservation facilitates the collection and long-term storage of semen from genetically superior males (5). This permits the widespread, efficient use of valuable genetic lines to rapidly improve herd or flock characteristics (e.g., disease resistance, yield) (6). Sperm cryopreservation is a pivotal technology that noticeably enables the distribution and implementation of artificial insemination (AI) across diverse livestock sectors (7). Thus, AI utilizing cryopreserved semen is essential for improving the genetic merit and production efficiency of rabbit (8). While sperm cryopreservation is a valuable tool in livestock breeding management, its success is limited by several challenges (9), primarily cryoprotectant toxicity and elevated oxidative stress, both of which compromise the fertilizing capacity of the spermatozoa (5).

Rabbit spermatozoa are a relevant model for cryopreservation procedure development because they respond poorly to glycerol, a major component in many standard cryopreservation protocols. This poor response mimics the challenge faced by other cell types for which glycerol is not an effective cryoprotectant (CPA) (10). However in rams (11) and buffaloes (12), the use of glycerol at low levels show positive effects on sperm quality and cryo-resistance. Conversely, supplementing the freezing media with sucrose (SU) or trehalose (TR) significantly improved sperm quality after cryopreservation. Cryoprotectants are chemicals designed to prevent cell injury during freezing. Glycerol (GL) or dimethyl sulfoxide (DMSO) are the main conventional penetrating cryoprotectant used across many species (11, 13, 14). However, a significant drawback is its cytotoxicity, which negatively affects sperm function and biological integrity. This detrimental effect has been documented in various species. The optimal concentration of DMSO established for cryopreservation in rabbits was 6% (15), which yielded favorable outcomes regarding sperm motility and viability. However, the effect of using sub-optimal or lower DMSO concentrations has not yet been adequately investigated. Conversely, another study suggested that DMSO can induce mitochondrial dysfunction and impair mitochondrial morphology (16); however, this effect may be contingent upon the use of elevated concentrations (17). Unfortunately, this level has been pointed out to be negative to rabbit sperm functionality and structure, significantly lowering fertility rates. The toxicity associated with high GL concentrations is likely due to osmotic stress (18). Consequently, there is a critical need to refine cryopreservation protocols by reducing the standard glycerol load or comparative with DMSO when fortified with sucrose or trehalose. Disaccharide sugars, notably SU and TR, function as effective extracellular cryoprotectants because they are unable to permeate the cell membrane. Recent research highlights the benefits of incorporating these sugars into sperm freezing protocols across different species. A research by Rostami et al. (19) demonstrated that adding SU (60 mM), and TR (100 mM) in combination with vitamin E (2 mM), to a Tris-egg yolk extender substantially increased the superiority of ram sperm after post-thawing. Research by Suksai and Dhanaworavibul (20) indicated that the inclusion of TR (50 mM) improved the quality of human sperm after thawing. In addition, Thananurak et al. (21) found that the use of SU had a significant positive impact on the quality and *in vivo* fertility of cryopreserved chicken sperm. Due to the discrepancies in the above results, we designed this experiment to re-evaluate the cryoprotective effects of DMSO at reduced doses, comparing it with glycerol when used individually or fortified with the disaccharide sugars sucrose (SU) and trehalose (TR) in rabbit sperm freezing media. The purpose of this experiment was to investigate the individual and combined effects of DMSO (with SU or TR) or glycerol on various key parameters, including sperm quality, sperm kinematics, apoptotic sperm, acrosome integrity, semen microbiota, cellular damage markers, and *ex vivo* fertility trial in rabbits.

## 2 Materials and methods

### 2.1 Ethical declaration

The inquiry was completed at the Faculty of Agriculture, Department of Animal Production, Mansoura University, Egypt. Ethical approval was granted by the Scientific Research Ethics Committee of Zagazig University under ethical code ZU-IACUC/2/F/223/2025, and all procedures involving animals adhered to the standards of the European Directive 2010/63/EU (22/09/2010) for husbandry and experimental handling. The study adhered to ARRIVE guidelines 2.0 to ensure animal welfare.

### 2.2 Bucks’ management and semen collection

For this experiment, 12 male New Zealand White rabbits served as sperm donors. They were 11–12 months old and had an average weight of 2.75 ± 0.25 kg. All bucks were fed a constant diet that was both isocaloric (2,700 kcal/kg) and isoproteic (13% protein), and water was freely available. Each rabbit was individually housed in a cage (60 × 40 × 35 cm^3^) with an automatic nipple drinker. The housing facility was an open-system farm exposed to natural environments (6). Veterinary checks were routinely performed to ensure the animals were free of pathogenic infections.

Following the procedure of Boiti, and Castellini (22), sperm samples were collected weekly from all 12 male rabbits using a rabbit artificial vagina (41–43 °C) and a live doe as a teaser. Semen collection was performed between 08:00 and 10:00 a.m., prior to the morning feeding. For ejaculates containing gel, raw semen samples were obtained after the gel was removed with forceps. Eighty-four ejaculates were obtained from 12 bucks during a seven-week collection period. Following collection, the semen ejaculates were analyzed for volume (using a graduated tube), initial motility (using a phase contrast microscope), and sperm cell concentration (using a Neubauer hemocytometer). For inclusion in the study, ejaculates were required to possess the following features: white color, ≥0.2 mL volume, ≥ 70% (initial motility, and viability), ≤ 15% sperm abnormality, and ≥100 × 10^6^/mL sperm concentration cells. All semen collected on a given day was first pooled and then diluted.

### 2.3 Semen extension, equilibration, and freezing processes

According to the protocol published by Abdelnour et al. (6), the extender was prepared for cryopreserving rabbit semen. The extender was prepared using 100 mL of a Tris-based solution, which included 3.028 g/dL of Tris, 1.675 g/dL of citric acid anhydrous, and 1.25 g/dL of fructose, along with 20% (v/v) egg yolk. Additionally, it was enriched with antibiotics: penicillin at 100 IU/mL and streptomycin at 100 μg/mL. The basal Tris-based extender was supplemented with various cryoprotectants to form the following five experimental treatments:

- DM4: Tris-based extender + 4% dimethyl sulfoxide (DMSO).
- DMTR: Tris-based extender + 4% DMSO + 50 mM trehalose.
- DMSU: Tris-based extender + 4% DMSO + 50 mM sucrose.
- GL4: Tris-based extender + 4% glycerol.
- DMGL2: Tris-based extender + 2% DMSO + 2% glycerol.

The pooled expanded semen samples were mixed with one of the previous extenders. Dilution was performed at a 1:4 ratio (semen: extender). The diluted semen was then equilibrated by cooling it 5 °C over a 30-min period. The semen was then packaged into 0. 5 mL French straws (IVM Technologies, France). The straws were subjected to a 10-minute vapor phase cooling period by suspending them 4 cm above the surface of liquid nitrogen. The straws were cryopreserved in liquid nitrogen (−196 °C) for a duration of one month.

### 2.4 Sperm characteristics

The semen samples were assessed for progressive motility, viability, membrane integrity, and abnormalities at two-time points: after equilibrating at 5 °C for 30 min and following post-thawing at 37 °C for 30 s. For assessing progressive motility, a phase-contrast microscope (DM 500, Leica, Switzerland) with 100x magnification and a hot stage set at 37 °C was utilized. Progressive sperm motility was characterized as the ratio of sperm capable of moving forward in a long semi-arch pattern. A 10 μL aliquot of diluted semen was put on a pre-warmed slide and covered with a coverslip. The analysis was conducted blindly by the same professional investigator.

Sperm viability was assessed treating the dual eosin-nigrosin staining technique, as described by Moskovtsev and Librach (23). Semen smears were stained with a mixture of 5% eosin (vital stain) and 10% nigrosin (background stain). Live spermatozoa were identified as those that remained unstained by the eosin. The percentage of viable cells in each sample was established by counting 300 spermatozoa below high magnification (×400) utilizing a light microscope (Leica DM 500, Switzerland). Concurrently, morphological defects were evaluated on the same 300 sperm cells, primarily noting defects such as coiled, broken, terminally coiled, and bent tails.

The functionality and integrity of the sperm plasma membrane were considered using the Hypo-Osmotic Swelling (HOS) test, based on the procedure illustrated by Neild et al. (24). For the assay, 10 μL of semen was incubated with 100 μL of a hypo-osmotic solution for 30 min at 37 °C. This solution, with an osmolarity of 75 mOsmol/L, contained fructose (6.75 g/L) and sodium citrate (3.67 g/L). Following incubation, a drop of the mixture was examined under a phase-contrast microscope at 400× magnification. Spermatozoa exhibiting coiled or swollen tails—indicating an intact and functional membrane—were counted. The percentage of HOS-positive sperm was calculated from a total count of 300 sperm cells per slide.

### 2.5 Computer-assisted sperm analysis

Sperm motion physical features were quantified using Computer-Assisted Sperm Analysis (CASA), specifically the Sperm Vision analyzer (Ref: 12520/5000; Minitube, Tiefenbach, Germany), following the established protocol of Dessouki et al. (25). The system incorporated an Olympus BX microscope (Hamburg, Germany) connected to a rapid scan digital camera. Live sperm images were captured at 60 frames per second (60 Hz) using 4x dark-field illumination. The entire CASA system was maintained at 37 °C, and approximately 1,500 spermatozoa were analyzed per treatment group.

The motion classification of sperm was recognized, such as velocity average path (VAP, μm/s), linearity (LIN = VSL/VCL), distance curved line (DCL, μm), velocity curved line (VCL, μm/s), distance straight line (DSL, μm), distance average path (DAP, μm), wobble (WOB = VAP/VCL), beat cross frequency (BCF, Hz), straightness (STR = VSL/VAP), and amplitude of lateral head displacement (ALH, μm).

### 2.6 Assessment of sperm acrosome status

Acrosome integrity was assessed using the Giemsa staining technique. Frozen–thawed sperm samples (200 μL) were initially mixed with 0.2% trypan blue and incubated for 10 min at 37 °C. The suspension was then extended with 2 mL of modified medium and centrifuged at 700 × g for 6 minutes (26). The supernatant was discarded, and the sperm pellet was resuspended in mBO medium. This washing and resuspension process was repeated until the suspension exhibited a light blue color. Finally, a smear of the processed sperm suspension was prepared on a glass slide, stained with a 10% Giemsa solution, and examined under a light microscope.

The assessment criteria were as follows:

- Live sperm with intact acrosome: post-acrosome is white, and the acrosome is pink/purple.
- Dead sperm with intact acrosome: post-acrosome is blue, and acrosome is dark pink/purple.
- Live sperm with detached acrosome: post-acrosome is white, and acrosome is white.
- Dead sperm with detached acrosome: post-acrosome is blue, and acrosome is white/gray.

Approximately one hundred spermatozoa were at random designated per slide for inspection.

### 2.7 Assessment of apoptosis and reactive oxygen species (ROS) by flow cytometry

Apoptosis-like changes in frozen–thawed spermatozoa were evaluated using Annexin V staining and flow cytometry (27). Sperm from each group was centrifuged to isolate the cells under cooling centrifugation system. The resulting sperm pellets were resuspended in 2 mL of binding buffer and totally mixed. A 100 μL aliquot of the sperm suspension was then mixed with 5 μL of Propidium Iodide (PI), labeled with phycoerythrin, and 5 μL of Annexin V, labeled with fluorescein isothiocyanate (FITC). The mixture was incubated in the dark for 15 min. Following incubation, 200 μL of binding buffer was added to the suspension. To assess and quantify the apoptotic status of the spermatozoa, the stained suspensions were immediately analyzed by flow cytometry. The analysis was performed using an Accuri C6 Cytometer (BD Biosciences, San Jose, CA, USA) and its associated software (28). The proportions of positive or negative Annexin V (A−/A+), PI (PI-/PI+), and the double-positive cells were assessed. According to Peña et al. (29), spermatozoa were categorized into four classes:

- Viable cells: without fluorescence signal and membrane dysfunction (A-/PI-),
- Viable sperm cells with early apoptosis: viable cells labeled with Annexin V but without PI (A+/PI-),
- Sperm cells with apoptosis: dead cells labeled with Annexin V and PI and with damaged permeable membranes (A+/PI+), and
- Sperm cells with necrosis: dead cells labeled with PI without Annexin V and with complete membrane loss (A-/PI+). Lastly, within each group, the spermatozoal counts for each of the specified categories were determined and documented.

The DCF H2DCFDA fluorescent assay (Abcam, Cat No. ab113851) was performed to measure spermatozoa reactive oxygen species (ROS) levels according to the manufacturer’s instructions. Spermatozoa samples were washed in PBS (1×10^6^ cells/mL). The samples were then centrifuged at 300–500 x g for 10 min at 4 °C to remove debris. The intact cells were incubated with 10 μM H2DCFDA dye at 37 °C for 30 min in the dark to allow for cellular uptake and oxidation by intracellular ROS. After incubation, the samples were analyzed using flow cytometry on a BD FACSCanto II (BD Biosciences, San Jose, CA). Fluorescence emission was measured at 525 nm following excitation at 488 nm. Data analysis was performed using FlowJo v10 (BD Biosciences). Intact spermatozoa were gated based on scatter properties, and the population was categorized as ROS-positive or ROS-negative.

### 2.8 Total antioxidant capacity (TAC) and oxidative-related biomarkers

After thawing, sperm medium was acquired by centrifugation of the post-thawed semen (1,000xg for 10 min). The collected supernatant samples were then transported to a fresh tube and stored at −20 °C. Antioxidant capacity and oxidative-related biomarkers such as total antioxidant capacity (TAC), malondialdehyde (MDA), hydrogen peroxide (H_2_O_2_), and nitric oxide (NO) were evaluated in the post-thawed semen samples discussing to Koracevic et al. (30), Ohkawa et al. (31), Abdel-Khalek et al. (15), and Aebi (32), respectively following simple colorimetric methods. The required parameters were analyzed using a Spectro UV–Vis Auto spectrophotometer (Model UV-2602, manufactured by Labomed, USA).

For the assays, all industrial kits were gained from Biodiagnostic Company (Giza, Egypt) and were utilized strictly following the manufacturer’s established guidelines.

### 2.9 Semen microbiota

The microbiological quality of the post-thawed semen samples was assessed by enumerating two distinct bacterial groups using standard culture methods. The total bacterial counts (TBC) were determined by plating serially diluted semen samples onto nutrient agar medium (33). Plates were incubated at 37 °C for 24 h. Results were reported as colony-forming units (CFU) per milliliter. For assessing total *Coliform* Bacteria, the detection and enumeration of *Coliform* bacteria followed the specific methodology outlined in (34). Plates were incubated at 37 °C for 16–18 h prior to counting. All microbiological analyses were conducted using seven independent semen samples for each treatment group to ensure statistical reliability.

### 2.10 Fertility trial

One hundred and twenty-five multiparous New Zealand White female rabbits with a mean (± SD) age of 5.2 ± 0.3 months and a body weight of 3.5 ± 0.4 kg were included in the study. Prior to inclusion, all does underwent a standard clinical examination by a veterinarian to exclude pathological conditions that could affect fertility. The rabbits were then randomly allocated to five groups (*n* = 25 per group). Artificial ovulation was induced in all animals by intramuscular administration of Receptal (Intervet equivalent, B. V. Boxmeer, Holland) at a dose of 0.25 mL/doe, acting as a GnRH analog. Giving to Eschborn (35) procedure, AI was performed using disposable plastic curved pipettes (Imporvet, S. A., Barcelona, Spain).

The does were inseminated with 1 mL of frozen–thawed semen (75 × 10^6^) and deposited deep into the vagina (12 cm beyond the pelvic brim) (36). Between 12 and 14 days after insemination, pregnancy was confirmed by abdominal palpation. The conception rate was subsequently calculated using the following formula:

$\begin{array}{l} \text{Conception Rate}\;(\%)=\text{Number of Pregnant}\;\mathrm{Doe}\\ /\text{Total Number of Inseminated Does}/100 \end{array}$

Upon parturition (delivery), the total litter size at birth was recorded.

### 2.11 Statistical analysis

The homogeneity of variances and normality of data distribution for all numerical variables were evaluated using Levene’s test and the Shapiro–Wilk test, respectively. The influence of various extender supplementations on sperm parameters was analyzed using a One-way Analysis of Variance (ANOVA) (SAS, 2002). The Duncan test was utilized to evaluate the significant differences. Data are stated as the means ± SEM, and the statistical difference is considered at *p* < 0.05.

## 3 Results

### 3.1 Sperm quality following 30-min equilibration at 5 °C

The effects of various cryoprotectants in a Tris dilution on the quality of rabbit sperm after a 30-min equilibration at 5 °C are presented in Table 1. The Tris-based extender supplemented with 4% glycerol (GL4 group) yielded the lowest rates of progressive motility and viability in rabbit spermatozoa (*p* < 0.05) compared to the other treatment groups. There was no significant impact on progressive motility and viability in all groups (*p* > 0.05), except for the GL4 group. A low dose of DMGL2 and GL4 exhibited the greatest sperm abnormality compared to other treatments (*p* < 0.01). Membrane integrity was the lowest in GL4 group (*p* < 0.05), followed by DM4 group compared to other treatments (*p* < 0.01). No significant differences were observed in membrane integrity between the DM4 group and the remaining treatment groups (*p* > 0.05).

**Table 1:** Effects of different cryoprotectants in a Tris-based extender on the quality of rabbit sperm following 30-min equilibration at 5 °C (Mean ± SE, *n* = 7).

Treatment	Progressive motility (%)	Viability (%)	Membrane integrity (%)	Abnormality (%)
DM4	81.4 ± 1.43^a^	83.3 ± 1.55^a^	82.4 ± 1.77^ab^	4.0 ± 0.53^b^
DMTR	83.6 ± 1.43^a^	86.7 ± 1.02^a^	84.4 ± 1.45^a^	5.1 ± 0.40^b^
DMSU	84.3 ± 1.30^a^	86.4 ± 1.00^a^	86.3 ± 1.30^a^	5.0 ± 0.44^b^
GL4	75.0 ± 1.54^b^	78.4 ± 1.17^b^	76.9 ± 1.42^b^	7.1 ± 0.74^a^
DMGL2	82.1 ± 1.01^a^	85.7 ± 0.87^a^	83.3 ± 0.94^a^	7.0 ± 0.44^a^
*p* value	0.0003	<0.0001	0.001	0.001

Distinct symbols (^a,b^) within the same column indicate significant variations between treatments (*p* < 0.05). The Tris-based extender was fortified with 4% DMSO (DM4), 4% DMSO + Trehalose 50 mM (DMTR), 4% DMSO + Sucrose 50 mM (DMSU), Glycerol 4% (GL4), or 2% DMSO + 2% Glycerol (DMGL2).

### 3.2 Sperm quality and kinematic features of post-thawed rabbit sperm

The sperm kinetics of post-thawed rabbit sperm, such as DAP (μm), DSL (μm), VAP (μm/s), and VCL (μm/s), were not affected by the freezing media enriched with various cryoprotectants (Table 2). The fortified DMSU group yielded the highest PM values, which were followed sequentially by those of the DMTR group. The GL4 group had better DCL values than the DMGL2 group (*p* < 0.01), however, the other treatments did not produce significant effects (*p* > 0.05). VSL (μm/s), ALH (μm) and STR (%), were greater in DM4 and DMSU groups. BCF (Hz) was higher in the DMTR and GL4 compared to other treatments (*p* < 0.01). WOB (%) was the lowest in the GL4 group (*p* < 0.05), while LIN (%) was the lowest in the DMTR and GL4 groups (*p* < 0.05).

**Table 2:** The influence of several cryoprotective agents, added to a Tris-extender on quality and kinematic features of frozen–thawed rabbit sperm (Mean ± SE, *n* = 7).

Item	Treatment					*p* value
	DM4	DMTR	DMSU	GL4	DMGL2	
Viability (%)	46.0 ± 1.45^ab^	47.4 ± 1.02^a^	49.6 ± 1.81^a^	33.9 ± 0.83^c^	41.0 ± 1.27^b^	<0.0001
Membrane Integrity (%)	43.4 ± 1.11^a^	45.0 ± 1.09^a^	48.0 ± 1.86^a^	30.4 ± 0.81^c^	38.0 ± 1.20^b^	<0.0001
Abnormality (%)	6.6 ± 0.48^b^	6.7 ± 0.61^b^	9.1 ± 0.74^ab^	12.0 ± 1.00^a^	9.1 ± 0.59^ab^	<0.0001
Progressive motility (%)	44.2 ± 0.94^b^	45.2 ± 1.55^ab^	48.7 ± 0.93^a^	34.0 ± 0.97^c^	42.1 ± 0.64^b^	<0.0001
DAP (μm)	19.7 ± 0.59	21.2 ± 0.68	19.7 ± 0.70	20.4 ± 0.95	19.1 ± 0.91	0.40
DCL (μm)	32.8 ± 1.34^ab^	37.2 ± 1.29^ab^	34.7 ± 1.59^ab^	39.0 ± 1.41^a^	31.7 ± 1.98^b^	0.02
DSL (μm)	15.3 ± 0.27	13.5 ± 0.42	14.7 ± 0.36	13.4 ± 0.32	13.5 ± 0.85	0.07
VAP (μm/s)	50.3 ± 1.54	52.9 ± 2.11	50.3 ± 1.61	47.9 ± 2.37	49.1 ± 2.36	0.52
VCL (μm/s)	82.6 ± 3.63	91.7 ± 4.00	87.1 ± 3.57	90.0 ± 3.85	80.6 ± 5.05	0.27
VSL (μm/s)	39.4 ± 0.78^a^	34.0 ± 1.18^ab^	38.0 ± 0.89^a^	31.6 ± 1.11^b^	35.0 ± 2.17^ab^	0.002
STR (%)	77.8 ± 1.01^a^	64.0 ± 1.48^b^	75.3 ± 1.76^a^	65.8 ± 2.77^b^	70.7 ± 1.20^ab^	<0.0001
LIN (%)	47.3 ± 1.15^a^	36.7 ± 1.12^b^	43.5 ± 1.82^a^	34.8 ± 1.33^b^	43.2 ± 0.40^a^	<0.0001
WOB (%)	60.5 ± 1.31^a^	57.2 ± 0.70^a^	57.5 ± 0.99^a^	52.5 ± 1.28^b^	60.7 ± 0.99^a^	<0.0001
ALH (μm)	6.4 ± 0.15^a^	4.5 ± 0.22^bc^	6.2 ± 0.17^a^	3.8 ± 0.15^c^	5.3 ± 0.51^ab^	<0.0001
BCF (Hz)	14.0 ± 0.91^b^	23.2 ± 1.23^a^	14.1 ± 0.37^b^	23.8 ± 0.38^a^	16.3 ± 0.86^b^	<0.0001

PM, progressive motility (%); DAP, distance average path (μm); STR, straightness (VSL/VAP); DCL, distance curved line (μm); DSL, distance straight line (μm); VAP, velocity average path (μm/s); VCL, velocity curved line (μm/s); BCF, beat cross frequency (Hz) VSL, velocity straight line (μm/s); LIN, linearity (VSL/VCL); WOB, wobble (VAP/VCL) and ALH, amplitude of lateral head displacement (μm).

Distinct symbols (^a–c^) within the same row indicate significant variations between treatments (*p* < 0.05). The Tris-based extender was fortified with 4% DMSO (DM4), 4% DMSO + Trehalose 50 mM (DMTR), 4% DMSO + Sucrose 50 mM (DMSU), Glycerol 4% (GL4), or 2% DMSO + 2% Glycerol (DMGL2).

### 3.3 Acrosome integrity

The proportion of dead sperm with an intact acrosome did not show significant differences across the various cryoprotectants added to the Tris extender (*p* = 0.15) (Table 3). However, the Tris extender supplemented with the DMSU group yielded the highest number of live sperm with an intact acrosome compared to other treatments, except for the DMTR group. In contrast, GL4 was associated with the lowest values. Live sperm exhibiting a detached acrosome was least prevalent in the Tris extender supplemented with DM4, DMTR or DMSU groups. Notably, the GL4 group resulted in the highest precentage of dead sperm with a detached acrosome (*p* < 0.01).

**Table 3:** Effects of different cryoprotectants in a Tris-based extender on acrosome reaction of post-thawed rabbit semen (Mean ± SE, *n* = 5).

Treatment	Acrosome integrity status (%)			
	Live sperm with intact acrosome	Live sperm with detached acrosome	Dead sperm with intact acrosome	Dead sperm with detached acrosome
DM4	38.0 ± 0.71^c^	20.2 ± 0.58^a^	35.0 ± 0.71	6.8 ± 0.58^b^
DMTR	41.8 ± 1.02^ab^	17.4 ± 0.87^b^	35.0 ± 0.32	5.8 ± 0.37^b^
DMSU	45.0 ± 0.71^a^	17.2 ± 0.86^b^	33.4 ± 1.36	4.4 ± 0.51^b^
GL4	28.8 ± 0.86^d^	21.0 ± 0.71^a^	36.8 ± 1.24	13.4 ± 1.03^a^
DMGL2	38.6 ± 0.93^bc^	19.0 ± 0.71^a^	36.2 ± 0.66	6.2 ± 0.58^b^
*p* value	<0.0001	0.01	0.15	<0.0001

Distinct symbols (^a–d^) within the same column indicate significant variations between treatments (*p* < 0.05). The Tris-based extender was fortified with 4% DMSO (DM4), 4% DMSO + Trehalose 50 mM (DMTR), 4% DMSO + Sucrose 50 mM (DMSU), Glycerol 4% (GL4), or 2% DMSO + 2% Glycerol (DMGL2).

### 3.4 Antioxidant biomarkers in post-thawed rabbit semen

**Figure 1:**
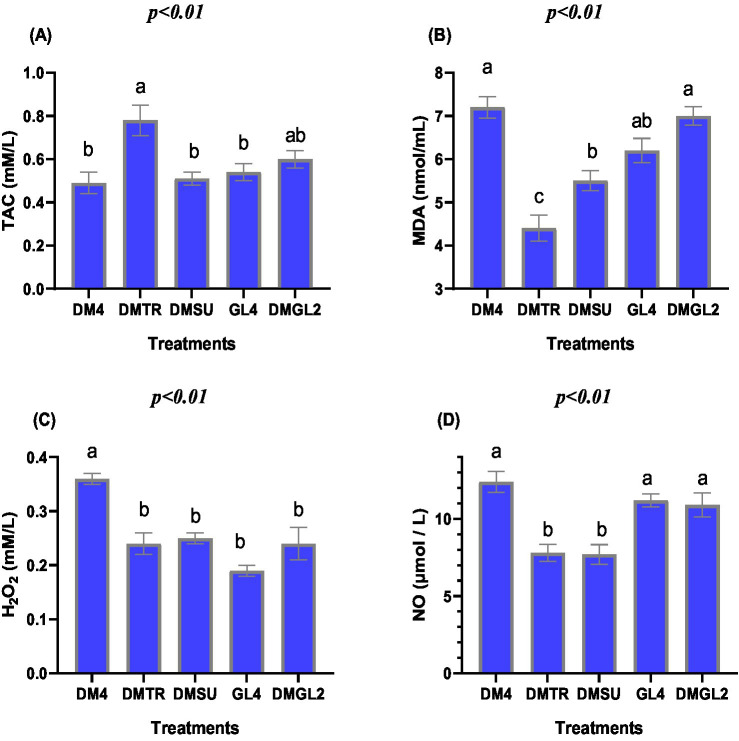
Effects of various cryoprotectants agents in a Tris dilution on total antioxidant capacity (TAC) **(A)**, oxidative-related biomarkers such as malondialdehyde (MDA) **(B)**, hydrogen peroxide (H_2_O_2_) **(C)**, and nitric oxide (NO) **(D)** in extender of post-thawed rabbit semen. Distinct symbols (^a–b^) indicate significant variations between treatments (*p* < 0.05, Mean ± SE, *n* = 5). The Tris-based extender was fortified with 4% DMSO (DM4), 4% DMSO + trehalose 50 mM (DMTR), 4% DMSO + surcose 50 mM (DMSU), glycerol 4% (GL4), or 2% DMSO + 2% glycerol (DMGL2).

As shown in Figure 1A, the DMTR group exhibited the highest total antioxidant capacity, although this difference was not statistically significant compared to the DMGL2 group (*p* > 0.05). MDA levels were significantly lower in the DMTR group compared to all other cryopreservation treatments (Figure 1B; *p* < 0.05), indicating a reduction in lipid peroxidation. The DM4 group exhibited the greatest levels of H₂O₂ (Figure 1C) compared to the other groups (*p* < 0.001), while the other semen treated samples did not differ for the elves of H₂O₂. The levels of NO (Figure 1D) were significantly lower in the DMTR and DMSU groups compared to all other experimental treatments (*p* < 0.01). There were no significant differences in NO concentrations among the GL4, DM4, and DMGL2 groups (*p* > 0.05).

### 3.5 Apoptosis-like changes (Annexin V/PI assay) and ROS

**Figure 2:**
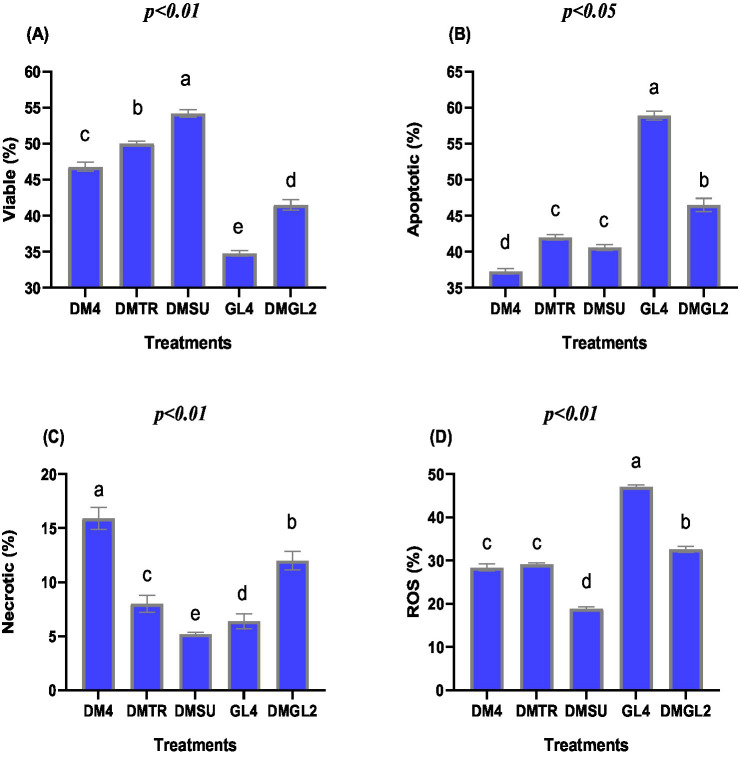
Effect of different cryoprotectants in a Tris-based extender on apoptosis-like changes (Annexin V/PI assay) involving viable **(A)**, apoptotic **(B)**, necrotic **(C)**, and ROS of post-thawed rabbit semen **(D)**. Distinct symbols (^a–e^) indicate significant variations between treatments (*p* < 0.05, Mean ± SE, *n* = 3). Th*e* Tris-based extender was fortified with 4% DMSO (DM4), 4% DMSO + trehalose 50 mM (DMTR), 4% DMSO + surcose 50 mM (DMSU), glycerol 4% (GL4), or 2% DMSO + 2% glycerol (DMGL2).

The DMSU group yielded the highest sperm viability, whereas the GL4 group exhibited the lowest values (*p* < 0.001; Figure 2A). The proportion of apoptotic spermatozoa was significantly higher in the GL4 group, whereas the DM4 treatment exhibited the lowest incidence of apoptosis (*p* < 0.05; Figure 2B). The DMSU treatment yielded the significantly lowest incidence of spermatozoal necrosis (Figure 2C), followed by the GL4 and DMTR groups, respectively (*p* < 0.001). In terms of ROS generation (Figure 2D), the DMSU group showed the lowest levels in post-thaw samples, followed by the DMTR and DM4 groups (*p* < 0.001). Conversely, the highest ROS concentrations were detected in GL4-treated semen samples (*p* < 0.001).

### 3.6 Post -thawed semen microbiota

**Figure 3:**
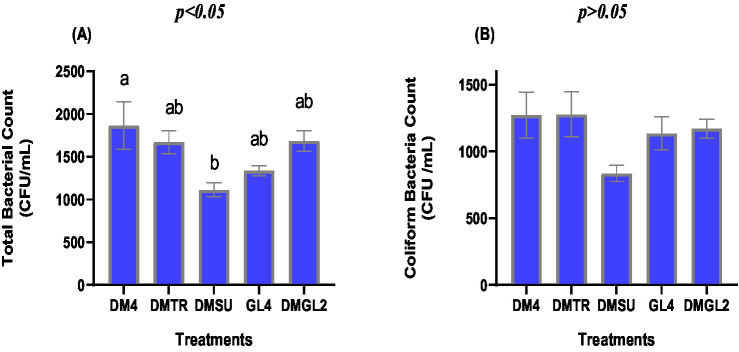
Effects of different cryoprotectants in a Tris-based extender on total bacterial count **(A)** and c*oliform* bacteria count **(B)** of post-thawed rabbit semen. Distinct symbols (a-b) indicate significant variations between treatments (*p* < 0.05, Mean ± SE, *n* = 7). The Tris-based extender was fortified with 4% DMSO (DM4), 4% DMSO + trehalose 50 mM (DMTR), 4% DMSO + sucrose 50 mM (DMSU), glycerol 4% (GL4), or 2% DMSO + 2% glycerol (DMGL2).

Although the DMSU group recorded the numerically lowest total bacterial count (Figure 3A; *p* < 0.01), no significant differences were observed when compared with the DMTR, GL4, and DMGL2 groups (*p* > 0.05). The total bacterial count was significantly lower in the DMSU group compared to the DM4 group (*p* < 0.015). The supplementation of the Tris-based extender with various cryoprotective agents had no significant effect on the *coliform* bacterial count (CFU/mL; *p* > 0.05; Figure 3B).

### 3.7 *Exo-vivo* fertility trial

**Figure 4:**
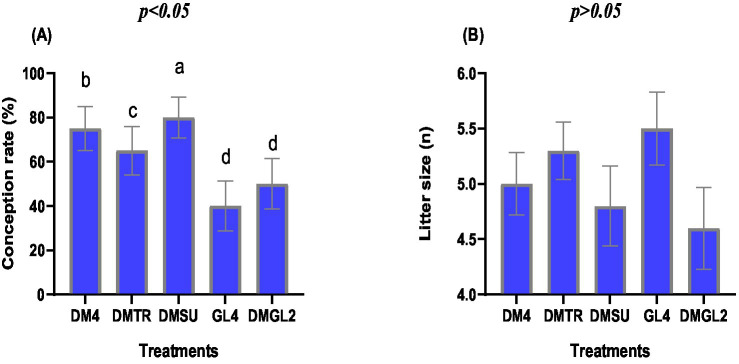
The conception rate **(A)** and litter size **(B)** of doe rabbits inseminated with semen extended different cryoprotectants in a Tris-based extender. Distinct symbols (^a–d^) indicate significant variations between treatments (*p* < 0.05). The Tris-based extender was fortified with 4% DMSO (DM4), 4% DMSO + Trehalose 50 mM (DMTR), 4% DMSO + Sucrose 50 mM (DMSU), Glycerol 4% (GL4), or 2% DMSO + 2% Glycerol (DMGL2).

The use of different cryoprotectants in a Tris-based extender significantly influenced the conception rate (Figure 4A, *p* < 0.05), while the litter size remained unaffected (Figure 4B, *p* = 0.37). The DMSU group achieved the highest conception rate (80.0%), followed by the DM4 group (75.0%) with statistical differences (*p* < 0.05). In contrast, conception rates reached their lowest levels in the GL4 and DMGL2 groups (*p* < 0.01).

## 4 Discussion

This study evaluates the efficacy of various rabbit semen cryopreservation media. Specifically, it compares the effects of low-concentration glycerol or DMSO both alone and in combination with non-penetrating cryoprotectants (sucrose or trehalose) on post-thaw sperm quality, kinematic parameters, apoptotic markers, and subsequent fertility outcomes, including conception rate and litter size. The results suggest that adding DMSO (4%) to extenders fortified with SU or TR can improve rabbit sperm quality after 30 min of equilibration at 5 °C and post-thawed semen compared to the Tris extender fortified with GL (2 or 4%). The supplementation of DMSO with either sucrose (SU) or trehalose (TR) led to significant improvements in post-thaw rabbit sperm quality. These enhancements were evident across several parameters, including reduced apoptosis-like changes, increased viability, improved plasma membrane and acrosome integrity, and superior progressive motility, kinematic traits, and conception rates compared to the other groups.

Progressive motility, viability, and plasma membrane integrity are very critical measures for accurately assessing sperm quality and the capability to fertilize an ovum (13, 20). The current experiment found that the freezing media fortified with DMSO (4%) to extenders fortified with SU or TR significantly heightened sperm quality, including progressive motility, viability, and plasma membrane function in rabbits. In contrast, the addition of glycerol may produce partially detrimental effects on these investigated parameters. Compared to 4% glycerol, the addition of 50 mM sucrose to DMSO-based media significantly enhanced sperm viability (12.5%), progressive motility (13.0%), and plasma membrane function (10.2%), while simultaneously decreasing abnormalities by 29.5%. Previous reports have demonstrated that the addition of 7% glycerol to cryopreservation extenders exerts detrimental effects on sperm function and ultrastructure in species such as the rabbit (37) and the buffalo (12). The observed cryoprotective benefits of DMSO are supported by previous studies (38), which found that its inclusion in freezing extenders protects effectively rabbit sperm viability and maintained sperm membrane function. Consistent with our findings regarding the superiority of DMSO over glycerol, previous reports indicate that a Tris-egg yolk extender supplemented with 3% DMSO yielded significantly higher sperm motility (54%) than the same extender containing 3% glycerol (47%) in rabbits (13). Preserving the integrity and stability of the sperm plasma membrane is essential for maintaining fertilizing potential and protecting genomic stability during cryopreservation. In contrast to our current findings, researchers found that 5% glycerol effectively preserves the semen of the local Vietnamese black rabbit by enhancing viability, motility, and membrane function (14). The differences in the effectiveness of glycerol and DMSO may be attributed to several factors, including the requirement for specific cryopreservation protocols tailored to each breed and variations in the fatty acid composition of the sperm membrane. This composition influences how effectively sperm binds with either DMSO or glycerol.

Given the limited studies comparing the effects of glycerol and DMSO on rabbit sperm functionality and quality, we have reviewed existing literature regarding other livestock species for comparison. While glycerol is established as a crucial cryoprotectant for maintaining the viability and survival of buffalo and cattle spermatozoa (12), its efficacy appears species-specific and may be less optimal for rabbits. Our findings align with Safaa et al. (39), who demonstrated that the combination of DMSO (3%) and sucrose (0.1 M) significantly improved motility and viability in rabbit semen. Conversely, in species such as rams (11) and buffaloes (12), low-level glycerol supplementation continues to exert positive effects on post-thaw sperm quality and function.

Conversely, supplementing the freezing media with sucrose or trehalose significantly improved sperm quality after cryopreservation. Sucrose (SU) exhibits significant cryoprotective properties, proven effective in freezing human sperm by minimizing harmful outcomes. A report by Awad (40) reported that applying 3% glycerol approved beneficial findings in terms of total, progressive motility and viability. Previous studies have shown that supplementation with trehalose and sucrose offers protective benefits to the sperm plasma membrane, improves overall cell viability, and reduces oxidative stress by lowering MDA levels (41). In contrast to our current findings, glycerol was found to be toxic to stallion spermatozoa at dilutions equal to or greater than 3.5%, primarily causing damage to the sperm membranes (41). These results are consistent with our current data. Computer-assisted sperm analysis (CASA) systems provide further insights into velocity categories, including straight-line velocity, curved line velocity, and average path velocity. The current findings are consistent with previous research indicating that the addition of 45 mM trehalose and 5% glycerol to the freezing media successfully enhanced buffalo sperm kinematics (17). In a previous study with buffaloes, Khalil et al. (12), found that adding 50 mM trehalose and a low level (3%) of glycerol significantly improved sperm kinematics. In rabbits, it was demonstrated that freezing media with DMSO improved sperm kinetics more than glycerol (42). Studies suggest that high concentrations of glycerol, such as 6%, have a negative impact on the fine motion ratio of post-thawed buffalo sperm by reducing linear motility and increasing circular motility (12). Importantly, there is no existing research in rabbits that has evaluated the combined effect of DMSO with sucrose or trehalose on sperm kinematic variables. However, the current results indicate that the improved rapid velocity after thawing is closely associated with enhanced plasma membrane functionality, suggesting a consistent protective effect of cryoprotectants on rabbit spermatozoa plasma membrane post-thawing. These detrimental changes are likely due to glycerol-induced toxic and/or osmotic shocks to rabbit sperm, a mechanism also observed in ram studies (19).

The cryopreservation process induces oxidative stress by promoting ROS production. This biochemical shift is characterized by increased levels of H_2_O_2_, NO, and the lipid peroxidation marker MDA, alongside a compensatory or depleted response in the antioxidant defense system (TAC). The DMTR group exhibited the highest levels of TAC, which coincided with a significant reduction in H_2_O_2_, NO and MDA compared to the other group. By leveraging its antioxidant properties, DMSO creates a more favorable cellular environment, thus protecting mitochondria and potentially improving their functional efficiency (43). A significant increase in ROS was observed in the GL4 group, while the lowest levels were found in the DMSU group (*p* < 0.05). Higher ROS concentrations were associated with a notable decline in both sperm viability and subsequent conception rates (80%). As a non-permeant cryoprotectant, sucrose stabilizes the plasma membrane and provides an osmotic environment that minimizes lipid peroxidation (19, 20). By maintaining cellular integrity during freezing, it effectively suppresses ROS generation and preserves post-thaw motility. The cryoprotective efficiency of sucrose stems from its ability to facilitate controlled cellular dehydration. By inducing an osmotic efflux of water prior to ice crystallization, it prevents mechanical membrane rupture and the subsequent release of ROS-generating enzymes into the medium (21, 41).

Acrosome integrity was higher in rabbit semen preserved with the addition of DMSO (3 M) and sucrose (0.1 M) (39). Furthermore, the current experiment demonstrated that the freezing extender fortified with DMSO (4%) and sucrose (50 mM) or trehalose (50 mM) exhibited a higher percentage of live sperm with intact acrosomes compared to other groups (*p* < 0.05). Similar results have been reported in buffaloes, where the addition of sucrose (50 mM) or trehalose (50 mM) to the GL extender significantly improved acrosome integrity (12). While glycerol and DMSO are the most common permeable cryoprotectants, glycerol is generally considered unsuitable for rabbit sperm preservation, potentially due to its low water permeability and high activation. Furthermore, high concentrations of DMSO are known to exert adverse effects on sperm quality, particularly concerning motility and acrosome integrity.

Apoptosis triggered during cryopreservation involves a cascade of cellular events, including caspase activation, alterations in mitochondrial membrane potential, and DNA fragmentation. Evaluating these apoptotic changes via flow cytometry represents a valuable approach for optimizing semen cryopreservation protocols. In this study, the DMSU group exhibited the highest percentage of viable sperm and the lowest rate of necrosis, which is consistent with the significant reduction in ROS levels observed in this treatment. This improvement in sperm viability and the subsequent reduction in necrotic cell percentages are attributed to the ability of sucrose to stabilize plasma membranes (12, 39). By acting as a non-permeant extracellular cryoprotectant, sucrose maintains cellular quality and mitigates osmotic stress, thereby indirectly reducing the generation of ROS (12, 20). Several studies highlight the positive role of sugar and glycerol supplements in cryopreservation. For example, Iqbal et al. (44) demonstrated that adding glycerol alone or with trehalose reduced the premature acrosome reaction in cryopreserved buffalo spermatozoa exposed to lysophosphatidylcholine. Independently, Thananurak et al. (21) found that sucrose supplementation in cryopreserved chicken sperm was superior to raffinose, resulting in a higher fertility rate (91.16% *vs*. 70.38). Our results indicate that the DMSO combinations yield more positive effects on sperm quality, kinematic parameters, and reduction of oxidative stress than glycerol alone.

To confirm the *in vitro* results, *ex vivo* (or *in vivo*) fertility trials are necessary, as an enhanced conception rate directly reflects improved reproductive efficiency in rabbit farming. In the present study, the DMSU group achieved the highest conception rate (80.0%), followed by the DM4 group (75.0%) with statistical differences (*p* < 0.05). Consistent with our current findings, previous research reported a conception rate of 60% when using a cryopreservation medium supplemented with 3% DMSO, compared to 50% for medium containing 3% glycerol (13). These consistent results align with the overall findings of this research. Moreover, similar to our results, the study by Safaa et al. (39) found that adding DMSO (3 M) and sucrose (0.1 M) to the freezing media of rabbit semen resulted in improved fertility and a higher total born (litter size). In contrast with our results, Iqbal et al. (17) found that adding 45 mM trehalose, 15% egg yolk, and 5% glycerol to the extender improved *in vivo* fertility of buffalo bull spermatozoa.

Investigating the post-cryopreservation semen bacteria is critical, given that microorganisms significantly influence sperm quality and fertility (12). In the current study, while total bacterial counts varied significantly across experimental groups, coliform counts remained statistically consistent. Because cryopreservation alters both cellular integrity and the native microbial landscape, characterizing the bacterial role in post-thaw function is essential. The observed improvement in mice sperm quality following DMSO treatment (45), may be partially attributed to its inherent antimicrobial properties. Furthermore, previous studies have demonstrated that the integration of nanoparticles can effectively reduce pathogenic bacteria within the semen microbiota of various animal species (46, 47). A key limitation was the inability to perform molecular pathway analysis comparing the effects of GL versus DMSO (and their combinations) on post-thaw sperm quality, a restriction necessitated by limited resources. We recommend that future studies explore the antioxidant status of the semen and identify the underlying molecular mechanisms to fully validate these results.

## 5 Conclusion

This study provides a reconsideration of adding cryoprotective agents such as glycerol and DMSO to rabbit semen after cryopreservation. Adding DMSO to freezing extender for rabbit semen alone or in combination with sucrose or trehalose enhanced sperm quality (progressive motility, membrane function, and viability) and improved acrosome integrity while reducing apoptotic sperm. Moreover, the total antioxidant status and total bacterial count were improved, while oxidative markers (MDA, H_2_O_2_, and NO) were reduced. The results of this study clearly highlight that the use of dimethyl sulfoxide (DMSO), or its combination with the disaccharides sucrose or trehalose, provides enhanced cryoprotection compared to glycerol in rabbit semen. This conclusion is based on the significantly improved semen quality parameters observed in the post-thaw samples. To validate these findings, future research should incorporate advanced omics technologies. Specifically, transcriptomic analysis (including microRNA and circular RNA profiling) and proteomics of the post-thawed sperm will provide deeper molecular insights into the observed cryoprotective effects.

## Data Availability

The raw data supporting the conclusions of this article will be made available by the authors, without undue reservation.
